# Optimization Study of a High-Efficiency Preservative for Ammonia-Free Concentrated Natural Rubber Latex

**DOI:** 10.3390/polym17020188

**Published:** 2025-01-14

**Authors:** Liguang Zhao, Peng Xing, Liyang Zhao, Qigui Yang, Yazhong Song, Li Ding, Tao Zhao, Yuekun Wang, Zhenxiang Xin, Hongxing Gui

**Affiliations:** 1 Hainan Natural Rubber Technology Innovation Center, Rubber Research Institute, Chinese Academy of Tropical Agricultural Sciences, Haikou 571101, China; zhaoliguangcatas@126.com (L.Z.); a23305805oo@163.com (P.X.);; 2 College of Polymer Science and Engineering, Qingdao University of Science and Technology, Qingdao 266061, China; 3 No. 4 Oil Production Plant, Changqing Oilfield Branch, China National Petroleum Corporation, Xi’an 710014, China; zly6_cq@petrochina.com.cn (L.Z.); yqg_cq@petrochina.com.cn (Q.Y.)

**Keywords:** concentrated natural rubber latex, ammonia-free, stability, mechanical properties

## Abstract

Ammonia is commonly used as a preservative in the production of concentrated natural rubber latex (CNRL) and latex products; however, it poses a serious risk to human health and the environment. In this study, we investigated a thioacetamide derivative (TD) as a preservative of ammonia-free CNRL and the optimization of a stabilization system comprising potassium hydroxide (KOH), lauric acid (LA), and sodium dodecyl sulfate (SDS) to enhance its preservation effect. The results revealed that an optimal amount of TD (0.03%) can effectively maintain the stability of CNRL, inhibit the increase in volatile fatty acid number (VFA number), maintain stable viscosity values, and improve the mechanical stability time (MST). However, increasing the TD dosage results in an increase in both the viscosity and VFA number and a decrease in MST. KOH was used to regulate the pH value of CNRL. It was also found that it can enhance considerably the mechanical properties of CNRL dry films and accelerates the vulcanization of vulcanized film; however, an excessive amount causes latex thickening. LA proved essential for improving the MST and reducing latex viscosity, thereby substantially enhancing the stability and processability of pre-vulcanized latex, but an excessive amount is detrimental to the curing speed and final mechanical strength. SDS can rapidly improve the MST and reduce the viscosity, but it negatively affects the surface molding of dry rubber films. In conclusion, KOH, LA, and SDS at appropriate dosages play a balancing and complementary role in the preparation of ammonia-free CNRL. Upon analyzing diverse performance metrics of CNRL, it has been determined that the optimal TD dosage ranges from 0.02 to 0.03% for maximum efficacy. The KOH dosage should be maintained within 0.1–0.15% to achieve the most favorable outcome, while the LA dosage is advisable to be kept between 0.06 and 0.1%.

## 1. Introduction

Natural rubber latex (NRL), an elastomer emulsion biosynthesized by plants, exhibits superior performance in diverse applications and is extensively utilized in impregnated, sponge, and press-out products [1]. NRL is predominantly composed of polyisoprene, the primary component responsible for its elasticity and strength. Additionally, it encompasses minor quantities of proteins, lipids, sugars, and other non-rubber substances [2]. Its abundance and environmentally friendly nature, coupled with its sustainability advantages, renders it superior to synthetic rubber [3]. However, NRL is highly vulnerable to microbial contamination under natural storage conditions [4]. The decomposition by bacteria and enzymes compromises the protective layer of rubber particles, generating fatty acids and reducing the interparticle forces, which causes rapid solidification and spoilage of NRL [5]. Bacterial action is the primary cause of latex spoilage, which can severely impact the quality of rubber and production efficiency. Therefore, preservatives that prolong the stability of latex without damaging the rubber structure and the processing and product performance must be added during the production process [6,7]. Currently, 0.7% ammonia is commonly used in production to preserve concentrated natural rubber latex (CNRL). Ammonia can adjust the pH value of the latex, exert an antibacterial effect, and neutralize acidic substances and complex metal ions to maintain latex stability [8]. However, ammonia is highly volatile, irritates the respiratory tract, and poses hazards to workers’ health [9,10]. Moreover, high concentrations of ammonia adversely affect the production environment of CNRL and latex products, leading to serious pollution problems. In line with sustainable development, the production of ammonia-free CNRL has become a research hotspot, aiming not only to minimize environmental risks but also to promote further development of the NRL industry [11].

Diverse substances, such as formaldehyde, urea, methylamine, hydroxylamine, sodium pentachlorophenol, boric acid, 8-hydroxyquinoline, streptomycin sulfate [12], and more recently, thiol-based benzothiazole, sodium alginate, tea polyphenols, modified chitosan, vegetable tannins, and ethanolamine, have been explored for NRL preservation [13,14,15], finding in some cases small-scale production application. For instance, in Malaysia, 2-[(hydroxymethyl)amino]ethanol is employed as a preservative in the preparation of ammonia-free CNRL [16]. Similarly, in China, sodium benzoate, sodium citrate, sodium tripolyphosphate, and other food preservatives are used to preserve ammonia-free CNRL. These preservatives, largely common chemical products in manufacturing, are typically used within the range of 0.1–1%. They exert a significant impact on the performance of the NRL. For instance, food preservatives and boric acid demonstrate poor preservation efficacy, while zinc oxide and benzoisothiazoline can cause latex thickening. Sodium pentachlorophenol presents high toxicity, and formaldehyde is carcinogenic. However, due to the varying effects of preservation, toxicity of the preservatives, and cost considerations, these preservatives have not yet reached large-scale application.

Ammonia-free CNRL colloids exhibit low stability, leading easily to increased thickening and solidification. These processed products demonstrate delayed precure rates, diminished wet gel strength, and suboptimal moldability [17]. These issues primarily stem from the CNRL preservation system, which comprises an alkali and a surfactant as stabilization components and an antimicrobial component including bactericidal agents, toxic enzymes, and mold inhibitors. The compromised stability of NRL can be enhanced by modifying the stabilization system with additional stabilizers. Furthermore, the reduced wet gel strength and prolonged vulcanization times, which are associated with high stabilizer concentrations in the latex [18], can be improved by regulating the stabilizer content. Concurrently, an antimicrobial system with superior preservation capabilities must be developed to enhance the application efficacy of ammonia-free CNRL.

The natural rubber processing team at the Rubber Research Institute of the Chinese Academy of Tropical Agricultural Sciences has a longstanding commitment to research on NRL preservation, having pioneered three distinct preservation systems based on Hexahydrohydroxyethyl triazine [19], Benzoisothiazolinone [20], and N,N′-Methylene-bis-Morpholine [21,22]. These systems can effectively preserve both fresh NRL and CNRL, leading to their practical application in production. However, the prohibitive cost of these preservatives has hindered their broader use. Meanwhile, the thioacetamide derivative (TD) has been found to effectively preserve NRL [23], with the added benefits of lower application cost, absence of volatile components such as formaldehyde, and environmental safety. The resulting low-ammonia CNRL exhibits superior overall performance and has proven beneficial in applications like latex pillows, mattresses, and balloons. Nonetheless, the paint and adhesive industries demand high environmental standards, for which the development of completely ammonia-free CNRL is required. In this study, aiming at overcoming the challenges faced by the preservation of ammonia-free CNRL using TD, which are associated with increased viscosity and compromised stability during storage, the stabilization system was optimized by modulating the preservative content to enhance the preservation efficacy of ammonia-free CNRL. This stabilization system incorporates potassium hydroxide (KOH), lauric acid (LA), and sodium dodecyl sulfate (SDS) as alkali and surfactant components, which allow modulating the stability of ammonia-free CNRL by adjusting their dosages, resulting in a suitable stabilization system for industrial production applications.

## 2. Experimental

### 2.1. Materials

NRL was sourced from the experimental farm of the Academy of Tropical Agricultural Sciences. The fungicide TD was prepared in a laboratory setting, for the preservation of NRL. Sodium carbonate, ammonia, glacial acetic acid, and ammonium sulfate were all analytically pure and procured from Guangdong Xilong Chemical Co. (Shantou, China). Concentrated sulfuric acid and barium hydroxide were also analytically pure and obtained from Guangdong Guanghua Science and Technology Co. (Shantou, China). KOH, zinc oxide (ZnO), sulfur, accelerator ZDC, SDS, and LA are commercially available rubber industry compatibility agents, as the NRL vulcanization agents.

### 2.2. Preparation of CNRL Samples

A specific quantity of fresh NRL was preserved using an ammonia-free preservation system, and ammonia-free CNRL was subsequently prepared through centrifugation in a continuous centrifuge. An appropriate amount of CNRL, as per the formulations outlined in Table 1 and Table 2, was taken and the corresponding preservatives (including ammonia, TD, KOH, SDS, and LA soap at a 10% solution) were added. The quantity of preservatives added was determined by the wet weight of the latex. After thorough shaking, the mixture was left to stand, respectively, and stored at room temperature.

### 2.3. Preparation of CNRL Dry Film

The concentrated natural rubber latex (CNRL) dry film samples are prepared refers to ISO 498: 1992.

### 2.4. Preparation of Vilcanlzed Film

Firstly, the appropriate amount of CNRL is placed in a clean beaker, and its concentration is diluted to 50% with deionized water. Secondly, the vulcanization agents are stirred and dispersed in the diluted CNRL under a water bath environment at 40 °C. Thirdly, pre-vulcanized latex is prepared by heating the above mixture to 60 °C, and then cooling and filtering. Fourthly, pour an appropriate quantity of pre-vulcanized latex into a clean glass dish and level it out, and then pre-vulcanized latex is dried at room temperature until it becomes transparent. Then, the film is immersed in deionized water for 24 h. Finally, vulcanized film is obtained by heating the film at 80 °C for 6 h until it turns translucent [22].

### 2.5. Preparation of Compound Film

**Figure 1:**
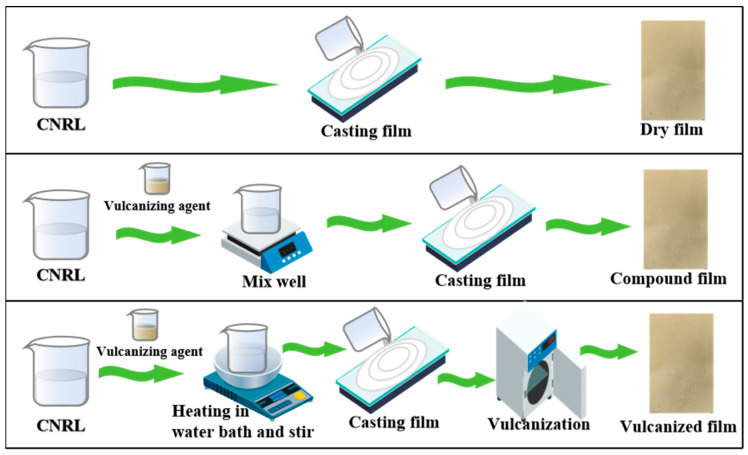
Preparation process of three kinds of concentrated natural rubber latex film.

Different from vulcanized film, the compound film is prepared by directly spreading the film after adding a vulcanization agent, stirring evenly, and then drying at room temperature. The detailed preparation process diagram is shown in Figure 1. The formula for the vulcanizing agent (dry basis, mass) is as follows: CNRL 100%, sulfur 1%, KOH 0.1%, peregal O 0.1%, ZDC 0.5%, and ZnO 0.4%.

### 2.6. Characteristics of CNRL

The volatile fatty acid value (VFA No.) test of CNRL refers to ISO 506:2020, the test of viscosity value of CNRL refers to ISO 1652:2011, the test of mechanical stability (MST) of CNRL refers to ISO 35:2004, and the determination of pH value of CNRL and pre-vulcanized CNRL refers to ISO23497:2019. The CNRL indicator is subjected to three tests and the results are subsequently averaged. Prior to testing, the latex must be heated to 35 °C by water bath.

### 2.7. Characteristics of Dry Film

The TENSOR 27 Fourier infrared spectroscopy tester (Bruker, Billerica, MA, USA) was employed for direct testing, with the detection range set between 4000 and 370 cm^−1^. The resolution was maintained at 4 cm^−1^, and the number of scans conducted was 32 times. The raw rubber samples were processed into particles, with 10 mg of each sample weighed and subsequently placed in a crucible. The thermogravimetric analysis was conducted using a STA449 model analyzer. The test parameters included an external atmosphere of nitrogen at a flow rate of 50 mL/min, high-purity nitrogen as the protective gas at a flow rate of 25 mL/min, a temperature range of 25 to 600 °C, and a temperature increase rate of 10 K/min. The molecular weight size and distribution of rubber were analyzed using Gel Permeation Chromatography (GPC). A 3 g sample of dry rubber was diced into fine strips and immersed in an adequate amount of tetrahydrofuran for a week until fully dissolved. The solution was subsequently filtered through a needle filter. Analysis was conducted at 30 °C using gel permeation chromatography.

### 2.8. Characteristics of Compound Film and Vulcanized Film

The vulcanization speed of a compound film was determined using the MD-3000A rotorless vulcanometer (Gotech Testing Machines Inc., Taichung City, Taiwan). The film’s thickness was approximately 4 mm, the temperature was maintained at 100 °C, and the determination process took 50 min. The tensile strength, elongation at break, constant tensile stress, and tear strength test for vulcanized rubber film samples refers to ISO 37:2017, and ISO 34-1:2015. Tensile strength test samples were dumbbell-shaped, with five parallel specimens prepared for each sample. The median value was recorded after conducting five tests. The tensile machine’s testing speed was set at 500 mm/min.

## 3. Results and Discussion

### 3.1. Preservative Effect of TD Dosage on CNRL

**Figure 2:**
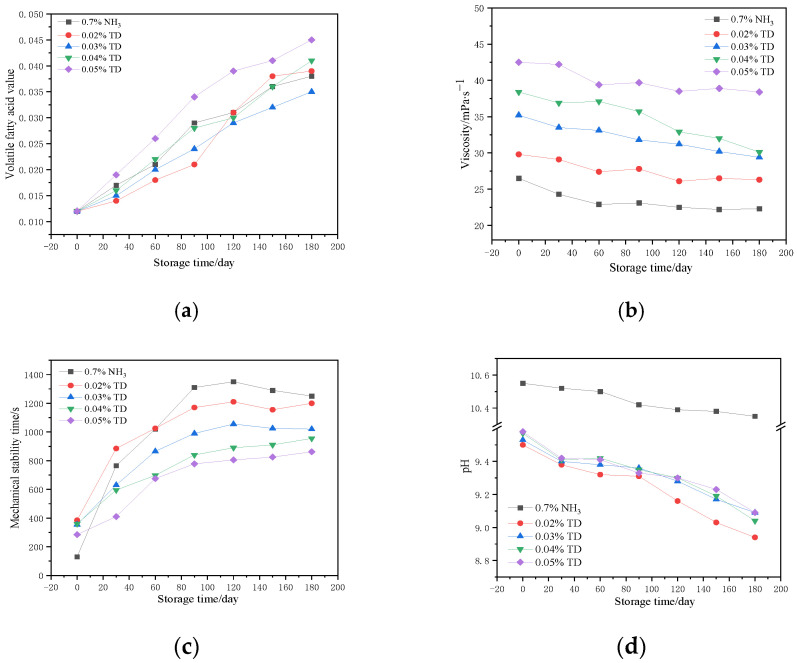
Changes in the properties: (**a**) volatile fatty acid number; (**b**) viscosity; (**c**) mechanical stability time; (**d**) pH of ammonia-free CNRL with different TD dosages.

Figure 2a illustrates the VFA number of CNRL over a period of 180 days with different dosages of the TD preservative. As depicted in Figure 2a, the disparity in VFA number between ammonia-free and high-ammonia CNRL (HA-CNRL) is minimal. The VFA number of ammonia-free CNRL with 0.05% TD was notably higher, with a more substantial increase with time. The VFA number of ammonia-free CNRL with 0.02–0.04% TD initially increased slowly, followed by a rapid increase in the later stages. In particular, the VFA number for 0.03% TD increased the most slowly and to the lowest extent. Figure 2b illustrates the variation in the viscosity value of CNRL over a period of 180 days with different dosages of the TD preservative. The results revealed a significant disparity in the viscosity values between ammonia-free CNRL and HA-CNRL. Specifically, the viscosity of HA-CNRL was consistently lower and more stable during the later stages of storage. The pattern of viscosity change in ammonia-free CNRL was similar to that of HA-CNRL, which means the viscosity decreased over time and stabilized in the latter stages. The quantity of TD exerted a clear influence on the viscosity value of ammonia-free CNRL; increasing the TD dosage resulted in a higher viscosity value. Conversely, reducing the amount of TD can effectively decrease the viscosity value of ammonia-free CNRL, thereby enhancing its fluidity. Figure 2c illustrates the changes in the mechanical stability time (MST) of ammonia-free CNRL with varying TD dosages over a period of 180 days. Ammonia-free CNRL and HA-CNRL exhibited similar patterns of change in their MST during storage, with both reaching values exceeding 650 s. Although the initial MST of HA-CNRL was lower, it increased at a faster rate and to a larger extent. Conversely, ammonia-free CNRL showed a higher initial MST but its increase was slower. The MST of ammonia-free CNRL was also influenced by the TD content. Specifically, an increase in TD dosage decreased the MST, which may be associated with changes in the VFA number of the latex [24]. The optimal MST of the latex was observed when the TD dosage ranged from 0.02 to 0.03%. Figure 2d shows the pH variation in ammonia-free CNRL with varying dosages of TD over a period of 180 days. The data revealed a significant disparity in the pH between ammonia-free CNRL and HA-CNRL. Specifically, the pH of HA-CNRL was higher and decreased less during storage. Conversely, ammonia-free CNRL displayed a lower pH value and experienced a more pronounced decrease during storage. Furthermore, the TD content exerted a discernible influence on the pH value of ammonia-free CNRL. An TD content of 0.02% led to the fastest decrease in pH down to the lowest value during storage.

### 3.2. Effect of the Stabilization System on Properties of CNRL

**Figure 3:**
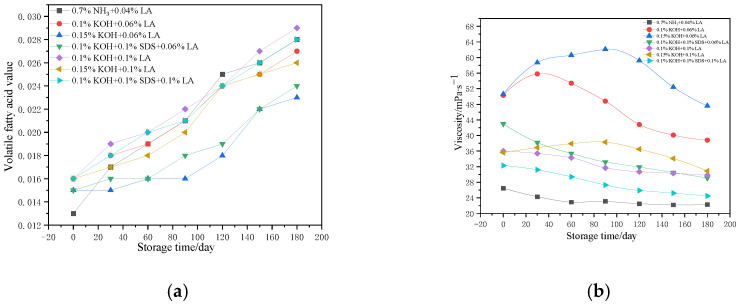

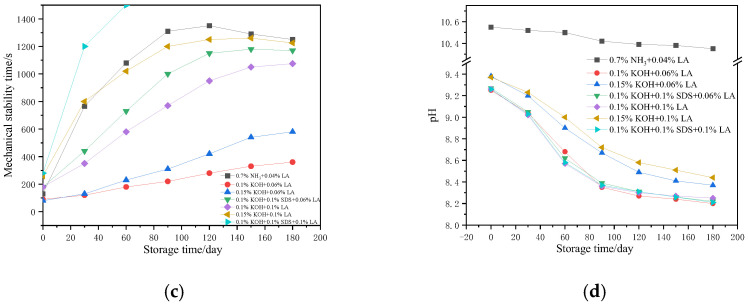
Changes in the properties: (**a**) volatile fatty acid number; (**b**) viscosity; (**c**) mechanical stability time; (**d**) pH of ammonia-free CNRL with different stabilization systems.

Figure 3a illustrates the fluctuations in the VFA number of both ammonia-free CNRL and HA-CNRL for various stabilization systems over a 180-day storage period. The VFA number of the seven CNRL samples experienced an upward trend during storage, albeit with variations in the rate and extent of this increase. Notably, HA-CNRL exhibited the lowest initial VFA number, but it showed a more rapid rate and higher extent of increase. Further analysis indicated that a higher KOH content correlated with a lower VFA number in CNRL, which was accompanied by a slower rate and lower extent of increase. Increasing the concentration of SDS allowed controlling the rate of increase in the VFA number of CNRL. Conversely, an increase in the LA content led to a higher VFA number in CNRL. Figure 3b displays the variations in the viscosity values of both ammonia-free CNRL and HA-CNRL for different stabilization systems over a 180-day storage period. The viscosity values of the seven CNRL samples consistently exhibited either a continuous decline or an initial increase followed by a decrease throughout the storage period. Notably, there were disparities in both the rate and extent of this decline. Specifically, the initial viscosity value of HA-CNRL was notably lower. Although it decreased during storage, this decrease was more gradual and less pronounced. For the six ammonia-free CNRL samples, the viscosity decreased with increasing dosage of LA and SDS. Conversely, an increase in the KOH dosage resulted in a higher viscosity. The viscosity reduction in CNRL was predominantly influenced by the surfactant dosage; an increase in this dosage can markedly diminish the latex viscosity. In this study, LA and SDS decreased considerably the latex viscosity. However, KOH may induce latex thickening; therefore, its dosage must be carefully controlled. Figure 3c illustrates the variations in the MST of ammonia-free CNRL and HA-CNRL during a storage period of 180 days. The MST of the seven CNRL samples demonstrated an upward trend during this period. However, there was a significant disparity in both the rate and magnitude of this increase. HA-CNRL exhibited a lower initial MST but a more rapid rate and a higher extent of increase. Further analysis indicated that the enhancement of the MST in CNRL primarily depended on the LA dosage. Although this effect took a considerable amount of time to manifest, the increase was relatively large. Conversely, SDS rapidly improved the MST of CNRL, but the increase was minimal. KOH also exerted some enhancing effect on the MST of CNRL, but its impact was relatively weak. Figure 3d shows the pH fluctuations of ammonia-free CNRL and HA-CNRL for various stabilization systems over a 180-day storage period. The pH of the seven CNRL samples consistently decreased during storage, albeit at varying rates and magnitudes. Notably, the initial pH of HA-CNRL was the highest, yet it exhibited the slowest rate of decline and the smallest overall decrease. In contrast, the initial pH values of the six ammonia-free CNRL samples were similar. Further analysis indicated that the pH of CNRL was predominantly influenced by the KOH concentration. Specifically, KOH markedly increased the pH of the latex, whereas LA and SDS exerted a minimal impact.

### 3.3. Effect of the Stabilization System on the Specific Conductance of CNRL

**Figure 4:**
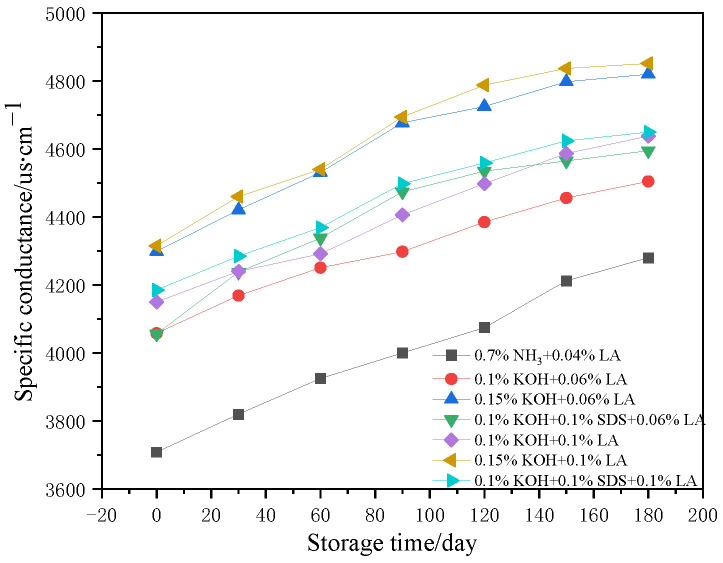
Specific conductance changes in ammonia-free CNRL with different stabilization systems.

Figure 4 illustrates the changes in the specific conductance of ammonia-free CNRL and HA-CNRL over a period of 180 days for various stabilization systems. The specific conductance of the seven CNRL samples experienced an upward trend during storage. Notably, HA-CNRL demonstrated the lowest initial specific conductance, which subsequently increased throughout the storage period, exhibiting a broad range of increases. The initial specific conductance of CNRL was predominantly influenced by the KOH amount. In contrast, LA and SDS had a negligible impact on the latex-specific conductance, which might explain why KOH induces latex thickening.

### 3.4. Effect of the Stabilization System on the CNRL Particle Size

**Figure 5:**
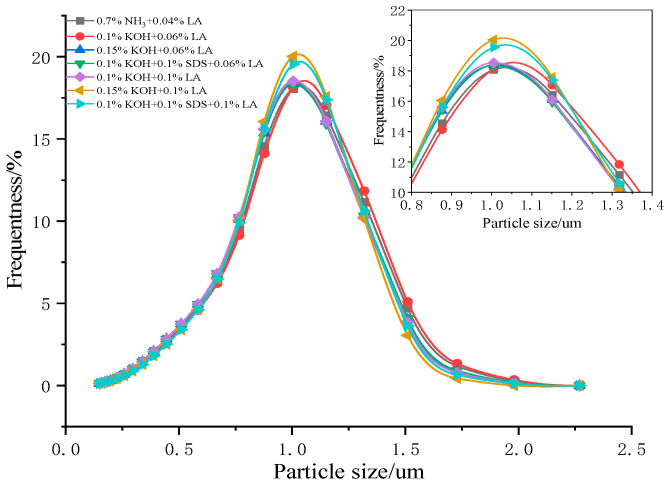
Particle size distribution of ammonia-free CNRL rubber with different stabilization systems.

Figure 5 and Table 3 present the particle size distribution and characteristic particle size values of CNRL for various stabilization systems in the absence and presence of high-ammonia concentrations in the rubber particles. A minimal variation in the particle size distribution was observed for the six ammonia-free CNRL samples and HA-CNRL.

### 3.5. Effect of the Stabilization System on the Physical and Mechanical Properties of CNRL

Table 4 summarizes the physical and mechanical properties of ammonia-free CNRL and HA-CNRL dry films for various stabilization systems. The results revealed a significant disparity in the physical and mechanical features between the six ammonia-free CNRL dry films and the HA-CNRL dry film. Specifically, the tensile strength, tear strength, hardness, and elongation stress of the HA-CNRL dry film were markedly superior to those of the ammonia-free CNRL dry films. Furthermore, distinct variations in the mechanical properties were observed among the six ammonia-free CNRL dry films. LA marginally decreased the tear strength, tensile strength, and elongation stress of the ammonia-free CNRL dry films. KOH was found to substantially enhance the mechanical properties of the dry films, including tensile strength, tear strength, and elongation stress. Similarly, SDS improved these mechanical properties to a certain degree; however, its effect was not consistent.

### 3.6. Effect of the Stabilization System on the Stability and Film Appearance of Pre-Vulcanized Latex

Table 5 presents the stability results for ammonia-free and high-ammonia pre-vulcanized latex for various stabilization systems. A significant disparity in the stability of these two types of latex was observed. High-ammonia pre-vulcanized latex exhibited relatively low MST, viscosity value, specific conductance, and a higher pH value. Although the film-forming surface of this high-ammonia CNRL was smooth, it underwent a substantial shrinkage. A notable variation in stability was observed among the six samples of ammonia-free pre-vulcanized latex. An increase in the LA content markedly enhanced the MST of the pre-vulcanized latex and reduced its viscosity value, resulting in minor wrinkles on the film surface. Increasing the KOH concentration marginally improved the MST of the pre-vulcanized latex but simultaneously increased its pH value and specific conductance, leading to increased viscosity and thickening as well as decreased thermal stability. The addition of SDS significantly boosted the MST of the pre-vulcanized latex and reduced its viscosity, albeit with a slight reduction in thermal stability. Increasing the dosages of KOH and SDS was detrimental to the flatness and smoothness of the film surface. It can be concluded that an excessive stabilizer dosage adversely affected the film’s flatness, resulting in uneven surfaces.

### 3.7. Effect of the Stabilization System on the Stability of CNRL

**Figure 6:**
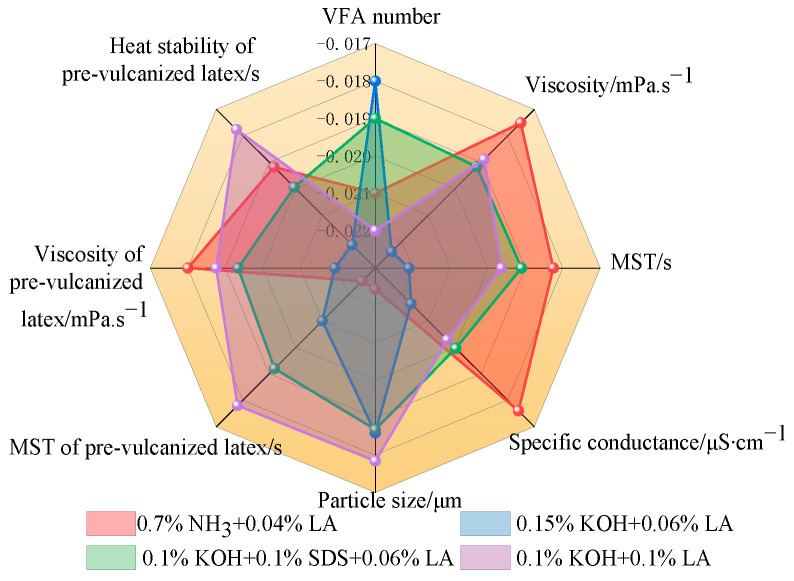
Statistical radar map of the stability indices of CNRL. Note: The mean value of VFA number, mean viscosity, and average particle size indices are plotted with their negative values.

**Figure 7:**
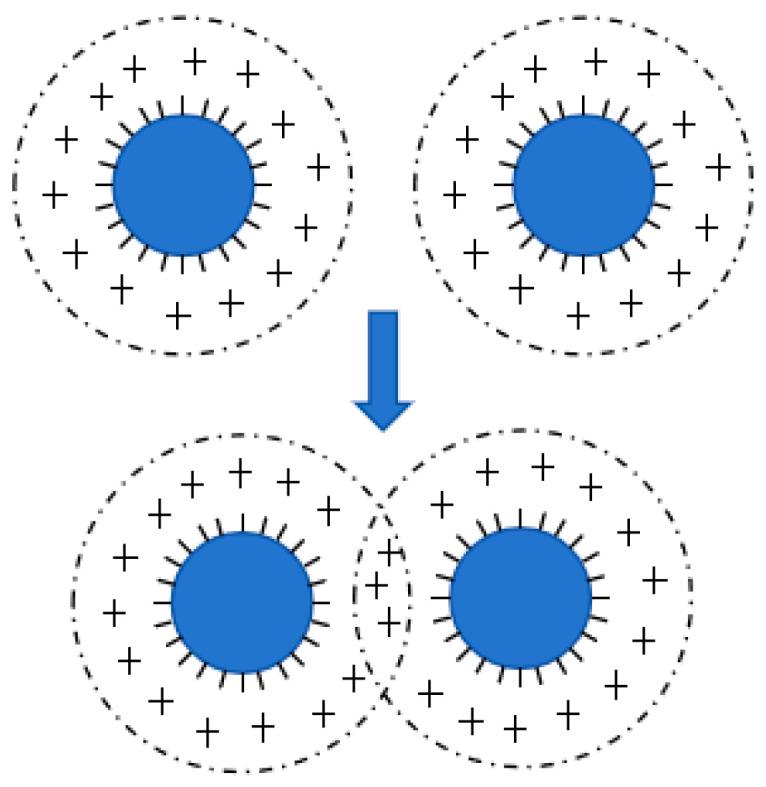
Rubber particle interaction diagram.

Figure 6 shows a statistical radar chart illustrating the impact of the stabilization system on the stability of CNRL. HA-CNRL exhibited superior MST, electrical conductivity index, and viscosity value before and after prevulcanization, thereby demonstrating relatively high stability. Although the presence of KOH was beneficial to the VFA number of ammonia-free CNRL, other indicators were comparatively low. CNRL with KOH displayed the highest VFA number index and precured latex heat stability index, along with optimal particle size. CNRL supplemented with SDS demonstrated good viscosity value, conductivity, and particle size index. Finally, CNRL supplemented with LA exhibited the highest viscosity index and optimal particle size for precured latex. The stability of NRL is primarily associated with the interaction force between the rubber particles (Figure 7) [25] and influenced by the rubber particle zeta potential [26]. Proteins on the surface of the rubber particles and adsorbed surfactants such as LA hydrolyze under alkaline conditions form –COO^−^, thereby rendering the surface of the rubber particles negatively charged. Simultaneously, a layer of positive charge is adsorbed at the periphery [27,28]. When the rubber particles are in close proximity, cation contact compression generates a repulsive force that inhibits the adhesion and fusion of the rubber particles, thereby maintaining the stability of the latex [24]. Therefore, the existence of electrolytes has a great influence on the stability of latex [29,30,31].

**Figure 8:**
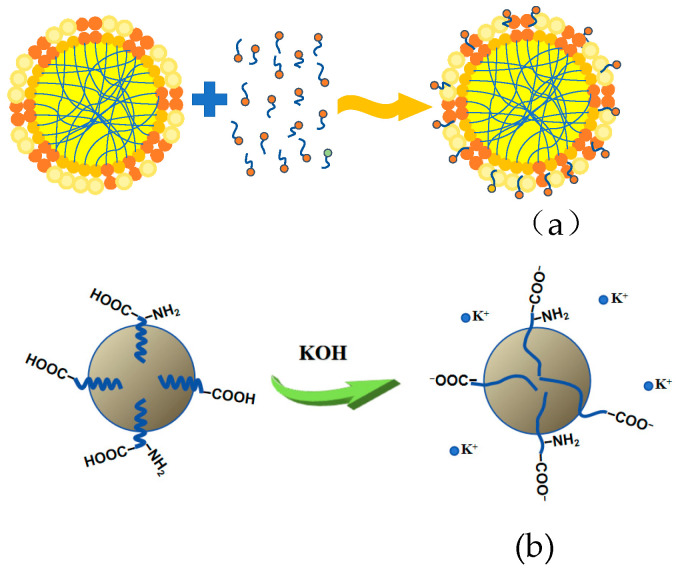
The change in the surfactant: (**a**) dispersion adsorption diagram; (**b**) surface surfactant ionization of rubber particles.

Figure 8a,b illustrate the adsorption of the surfactant on the surface layer of the rubber particles and the saponification reaction under alkaline conditions. The stability of the rubber particles is intimately linked to the interaction between the surfactant and KOH [32,33]. Surfactants, primarily long-chain fatty acids [27,28], can be uniformly dispersed in the solution after saponification under alkaline conditions. They remain in an ionic state under certain alkaline conditions, providing charge repulsion, increasing the zeta potential, and maintaining the stability of the latex [34]. Therefore, alkali and surfactants are essential for maintaining the stability of the latex [35,36]. Both KOH and ammonia can saponify fatty acids according to the following reactions:

NH_3_ + H_2_O ⇄ NH_4_^+^ + OH^−^ → R-COOH + NH_3_∙H_2_O ⇄ R-COO^−^ + NH_4_^+^ + H_2_O

R-COOH + KOH ⇄ R-COO^−^ + K^+^ + H_2_O

Since KOH is a strong base, the degree of the reaction is higher [37].

### 3.8. Vulcanization Characteristics of the Compounded Rubber Film

**Figure 9:**
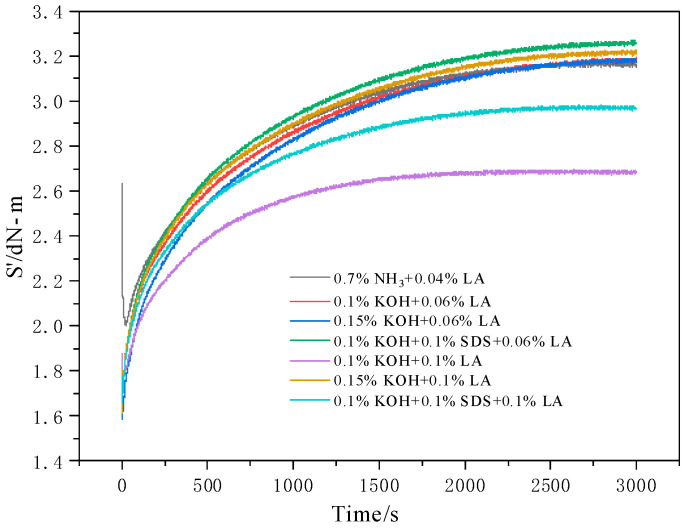
Vulcanization curves of compounded films with different stabilization systems.

Figure 9 presents the vulcanization curves for ammonia-free and high-ammonia compounded rubber films for various stabilization systems at 100 °C. The torque change rate (S′) of the seven rubber film samples consistently increased with extended vulcanization time. However, notable variations were observed in both the rate and overall magnitude of the increase. Specifically, the initial value of the vulcanization curve S′ for the high-ammonia compounded film was higher and showed a swifter rate of increase. The increase in S′ for the KOH-containing samples among the six ammonia-free compounded films was notably rapid, aligning closely with that observed for the high-ammonia compounded films.

Table 6 presents the vulcanization parameters for ammonia-free and high-ammonia-compounded films for various stabilization systems at 100 °C. The M_L_ values of the six ammonia-free compounded films were inferior to those of the high-ammonia samples, which can be attributed to the languid prevulcanization rate of ammonia-free latex. Concurrently, an increase in the LA dosage decreased the M_H_ and M_H_ − M_L_ values as well as the vulcanization rate of the composite film. In contrast, KOH and SDS increased these three parameters. A comprehensive analysis indicated that both KOH and SDS enhanced the vulcanization speed, with KOH having a more pronounced effect. In contrast, LA decelerated the vulcanization process. Furthermore, the TD preservative promoted the vulcanization of CNRL, substantially reducing the vulcanization time and improving the degree of vulcanization.

### 3.9. Characteristics of Vulcanized Rubber Films

Table 7 presents the physical and mechanical properties of vulcanized rubber films derived from various stabilization systems without ammonia, as well as those of HA-CNRL. The results revealed distinct variations in the physical and mechanical properties of the films. Notably, the vulcanized HA-CNRL film exhibited superior mechanical strength, as evidenced by its lower tensile stress and higher tensile strength and tear strength compared with those of vulcanized ammonia-free CNRL films. Furthermore, the mechanical properties of the six samples of vulcanized ammonia-free CNRL films were significantly different. For instance, the sample with KOH demonstrated a moderate level of tensile strength but the highest tear strength. Moreover, increasing the KOH concentration markedly enhanced the mechanical properties of the vulcanized rubber film. Meanwhile, the incorporation of SDS marginally boosted the tensile strength, albeit at the cost of a decrease in tear strength. Increasing the LA dosage affected negatively the mechanical properties, including tensile strength, tear strength, and elongation stress.

### 3.10. Infrared Curve of CNRL Dry Film

**Figure 10:**
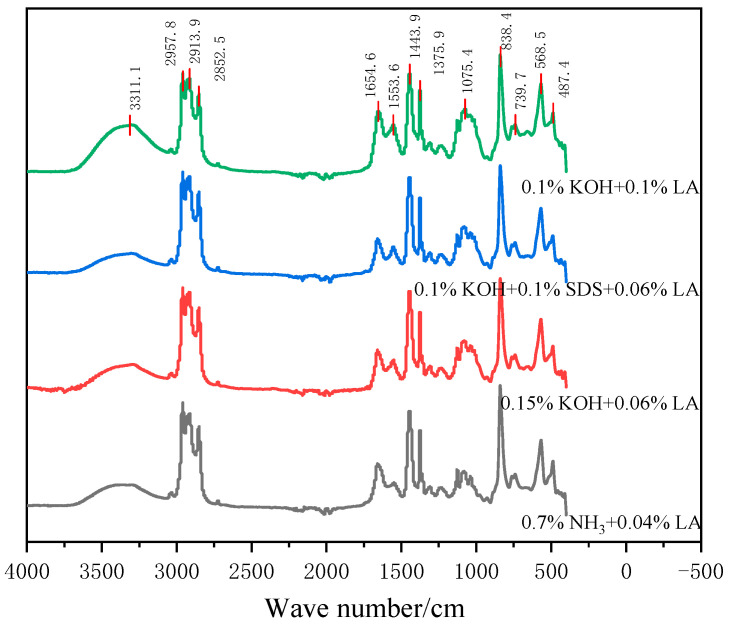
Infrared curve of CNRL dry films with different stabilization systems.

Figure 10 shows the IR spectra of four dry film samples of CNRL. It can be seen from Figure 10 that the spectra of CNRL samples without ammonia and HA-CNRL samples did not show significant wave peak migration, nor did the wave peak intensity changes significantly. The characteristic peaks appeared basically in the same wave number. The C=C double bond stretching vibration peak is 1654 cm^−1^ and the bending vibration peak is 838 cm^−1^. The stretching vibration peaks of -CH_3_ and -CH_2_ are 2957 cm^−1^ and 2852 cm^−1^, and their bending vibration peaks appear near the wave numbers of 1445 cm^−1^ and 1374 cm^−1^, respectively. The chemical structure of ammonia-free CNRL dry film samples was basically the same as that of high ammonia preserved. The preservative TD had no adverse effect on the structure of natural rubber.

### 3.11. Thermal Characteristics of CNRL Dry Films

**Figure 11:**
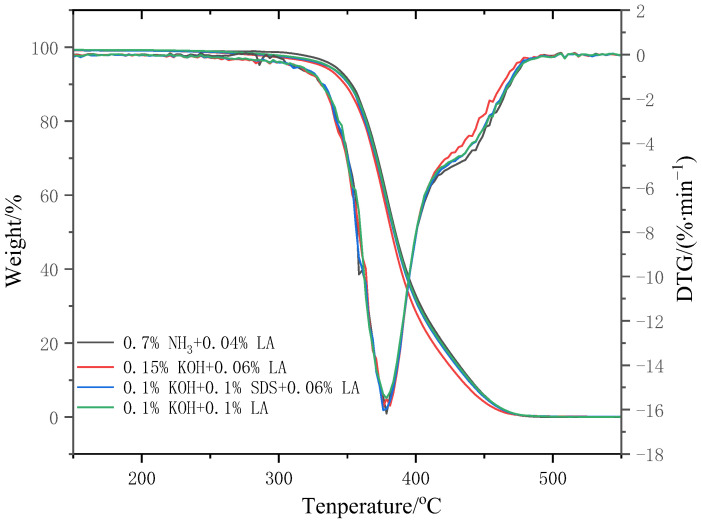
TG and DTG curves of CNRL dry films with different stabilization systems.

Figure 11 presents the thermogravimetry (TG) and Derivative Thermogravimetry (DTG) curves, respectively, for dry films of ammonia-free CNRL and HA-CNRL. Table 8 summarizes the characteristic temperatures associated with the thermal degradation of the dry gel film samples. The results revealed that the degradation curves for ammonia-free CNRL and HA-CNRL dry films were similar. The DTG curves for the four dry film samples exhibited an inconspicuous shoulder peak. Furthermore, the variations in the characteristic temperatures of the four samples were within the margin of error. Consequently, the dry film samples exhibited similar thermal stability. The stabilization system had a negligible impact on the thermal stability of CNRL, which is consistent with the high-ammonia preservation observed. Specifically, the stabilization systems exerted a minimal influence on the thermal stability of the ammonia-free CNRL films.

### 3.12. Molecular Weight Size and Distribution of CNRL Dry Films

**Figure 12:**
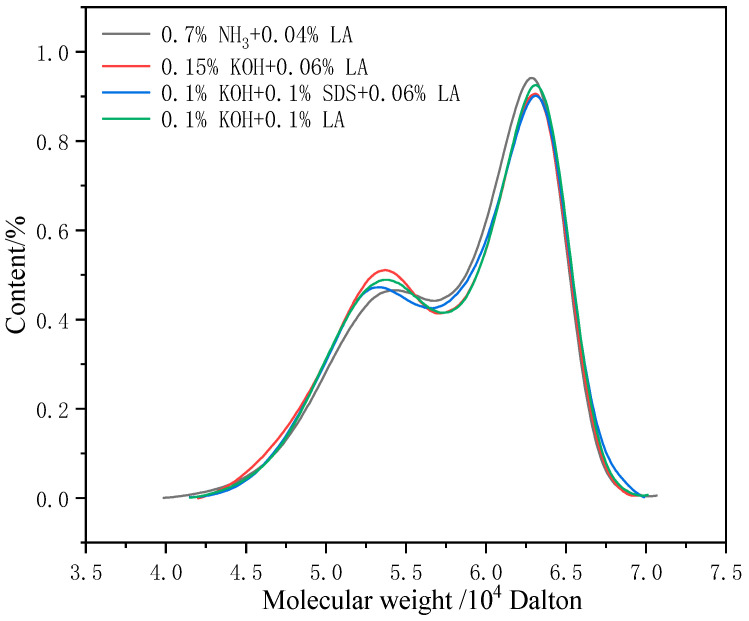
Relative molecular weight distribution of CNRL dry films with different stabilization systems.

Figure 12 shows the relative molecular mass distribution of selected CNRL dry film samples. Table 9 provides the relative molecular mass size and distribution coefficients of the corresponding dry film samples. As illustrated in Figure 12, the relative molecular mass distribution of the dry films exhibited a characteristic shoulder peak distribution. The molecular weight distribution images of the four dry film samples were consistent. Compared with the HA-CNRL samples, the high-molecular-weight fraction of the ammonia-free CNRL dry film samples was lower, whereas the low-molecular-weight fraction was higher. Concurrently, the peak shapes were broader and the molecular weight distribution coefficients were higher. The peak shape distribution of the four samples was essentially identical.

### 3.13. Effect of the Stabilization System on the Mechanical Properties of CNRL Films

**Figure 13:**
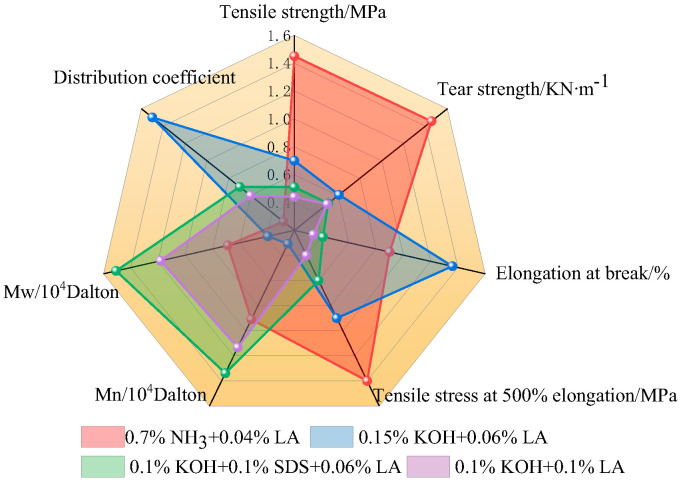
Radar map of the CNRL dry films.

**Figure 14:**
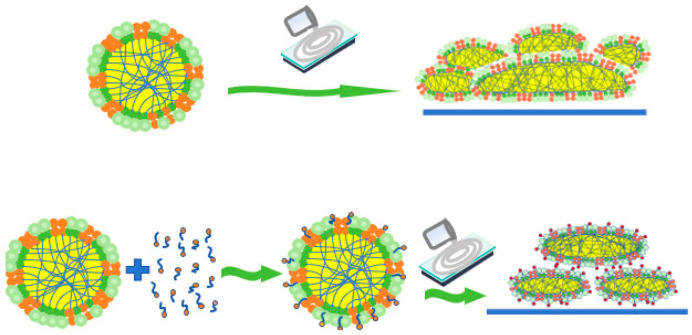
Surfactants inhibiting the fusion between rubber particles.

Figure 13 displays a radar chart depicting the physical and mechanical properties and the relative molecular mass of CNRL dry films. The HA-CNRL dry film exhibited superior tensile strength, tear strength, and 500% constant tensile stress. In the presence of KOH, the elongation at break and molecular weight distribution coefficient of the dry film were the highest. After SDS addition, both the number and relative molecular mass of the film reached their maximum values. However, all indices of the dry films deteriorated after LA addition. The addition of KOH resulted in the lowest mechanical properties of the dry films. This can be attributed to the ability of KOH to enhance the fatty acid saponification reaction and impede the fusion between rubber particles and intermolecular entanglement, thereby reducing the mechanical properties of the dry films. Upon adding SDS and LA, a thicker protective layer was formed around the rubber particles, which enhanced the stability and hindered the fusion between rubber particles (Figure 14), inhibiting the physical entanglement and crosslinking between the rubber molecules [38].

**Figure 15:**
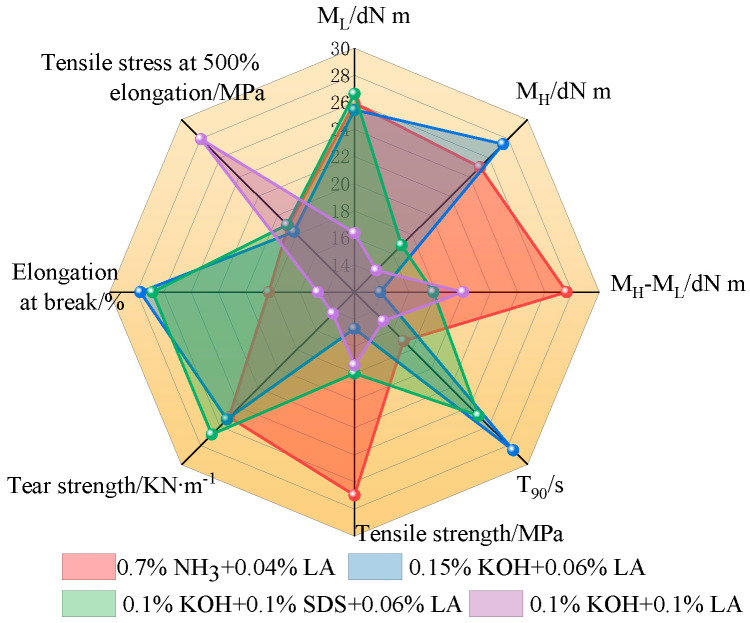
Radar chart of the composite film and vulcanized film. Note: T_90_ values are plotted using their negative values.

**Figure 16:**
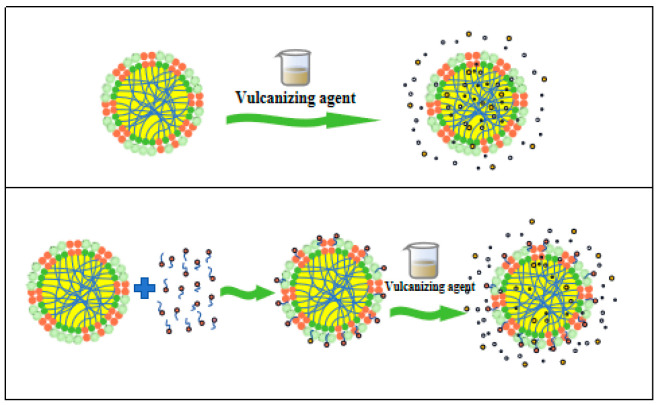
Surfactants inhibiting the diffusion of vulcanization agents.

Figure 15 presents the radar chart of the vulcanization coefficient for the CNRL films as well as the physical and mechanical property indices of the resultant vulcanized films. The HA-CNRL films exhibited superior torque differences and tensile strength. Upon addition of KOH, these films demonstrated enhanced torque M_H_ values, tensile strength, and elongation at break. Films treated with SDS exhibited elevated M_L_ values and tear strength, and those supplemented with LA displayed optimal elongation at break. Interestingly, vulcanized films of LA-supplemented ammonia-free CNRL exhibited notably reduced mechanical properties, likely due to LA inhibiting the diffusion of vulcanization agents and rubber particle fusion (Figure 16) [39]. In contrast, KOH improved considerably the mechanical properties and the film M_H_ value and reduced the T_90_ time, indicating a faster vulcanization rate [40]. It is worth noting that non-rubber alkaline substances such as choline, cholamine, and betaine, are known to promote vulcanization in natural rubber. In addition, the alkaline amino acids after protein hydrolysis on the surface of rubber particles can greatly improve the vulcanization rate of rubber [41].

The effects of alkali and surfactants on the vulcanization process and gel structure of NR latex films, as well as on the final physical and mechanical properties, are significant. Future research should conduct systematic studies to rigorously and comprehensively investigate the mechanisms of their impact on the structure of vulcanized NR gel films.

## 4. Conclusions

To optimize TD as a preservative of ammonia-free CNRL, the effect of a stabilization system comprising KOH, LA, and SDS on the preservation performance was investigated. The main conclusions can be summarized as follows:

(1) As the concentration of the TD preservative increases, both the VFA number and viscosity of ammonia-free CNRL increase progressively, whereas the MST decreases. Additionally, the pH value increases slightly. The optimal VFA number is observed at a TD content of 0.03%. Notably, when the viscosity value is reduced, the MST is enhanced, leading to superior preservation effects;
(2) KOH, LA, and SDS play distinct roles in the preparation and property optimization of CNRL. KOH primarily influences the pH and specific conductance of the latex; however, an excessive amount can result in latex thickening. LA is crucial for enhancing the MST and reducing the viscosity, and SDS improves the MST, reduces the viscosity, and suppresses the increase in VFA number. In practical applications, the selection and regulation of these additives should be adjusted according to performance indicators and process requirements;
(3) An increase in the LA dosage decreases marginally the tear strength, tensile strength, and constant elongation stress in the ammonia-free CNRL dry films, leading to enhanced mechanical and heat stability of pre-vulcanized latex. Additionally, it reduces the system viscosity, thereby facilitating the processing. However, an excessive LA content can result in minor surface wrinkles on the film, decreased curing speed, and reduced mechanical properties;
(4) The addition of KOH markedly enhances the mechanical properties of the dry films, specifically the tensile strength, tear strength, and elongation stress. It also marginally improves the MST. Furthermore, KOH increases both the pH and the specific conductance of the latex. The incorporation of KOH notably accelerates the curing speed and increases the mechanical properties of the resultant cured film. However, it simultaneously decreases the flatness and smoothness of the film surface, thereby affecting the overall quality of the film;
(5) SDS can enhance the tensile strength, tear strength, and constant tensile stress of the dry films to a certain degree. However, its effect is less stable compared with that of KOH. SDS significantly improves the MST of pre-vulcanized latex and effectively reduces the viscosity, which is beneficial for processing. Additionally, SDS accelerates the vulcanization process, albeit not as much as KOH. However, an increase in the amount of SDS reduces the surface flatness and smoothness, adversely affecting the film-forming properties. Although it enhances the tensile strength, it also decreases the tear strength;
(6) The CNRL dry films, regardless of the stabilization system used, exhibit stable molecular weight size and distribution, chemical construction, and thermal stability;
(7) From the analysis of various performance data of rubber latex, the TD dosage is maintained at 0.02–0.03% for the best effect, the KOH dosage is controlled at 0.1–0.15% for the most suitable, and the LA dosage is controlled between 0.06 and 0.1% for the most suitable. The appropriate dosage of SDS is between 0 and 0.1%.

## Figures and Tables

**Table 1:** CNRL preservation system ratios with different TD dosages.

Samples	NH_3_/%	TD/%	KOH/%	SDS/%	LA/%
0.7%NH_3_	0.7	-	-	-	0.05
0.02%TD	-	0.02	0.1	0.1	0.05
0.03%TD	-	0.03	0.1	0.1	0.05
0.04%TD	-	0.04	0.1	0.1	0.05
0.05%TD	-	0.05	0.1	0.1	0.05

**Table 2:** CNRL preservation system ratios with different stabilization systems.

Stabilization Systems	NH_3_/%	TD/%	KOH/%	SDS/%	LA/%
0.7%NH_3_ + 0.04%LA	0.7	-	-	-	0.04
0.1%KOH + 0.06%LA	-	0.03	0.1	-	0.06
0.15%KOH + 0.06%LA	-	0.03	0.15	-	0.06
0.1%KOH + 0.1%SDS + 0.06%LA	-	0.03	0.1	0.1	0.06
0.1%KOH + 0.1%LA	-	0.03	0.1	-	0.1
0.15%KOH + 0.1%LA	-	0.03	0.15	-	0.1
0.1%KOH + 0.1%SDS + 0.1%LA	-	0.03	0.1	0.1	0.1

**Table 3:** Particle size of ammonia-free CNRL particles with different stabilization systems.

Stabilization Systems	Average Diameter /μm	Pitch Diameter/μm	D_10_/μm	D_90_/μm
0.7%NH_3_ + 0.04%LA	0.8761	0.8895	0.4573	1.2581
0.1%KOH + 0.06%LA	0.8894	0.9025	0.4688	1.2702
0.15%KOH + 0.06%LA	0.8633	0.8766	0.4590	1.2358
0.1%KOH + 0.1%SDS + 0.06%LA	0.8636	0.8755	0.4607	1.2369
0.1%KOH + 0.1%LA	0.8608	0.8742	0.4601	1.2297
0.15%KOH + 0.1%LA	0.8647	0.8848	0.4793	1.2084
0.1%KOH + 0.1%SDS + 0.1%LA	0.8723	0.8892	0.4804	1.2269

**Table 4:** Mechanical properties of ammonia-free CNRL dry films with different stabilization systems.

Stabilization Systems	NH_3_/%	0.7	-	-	-	-	-	-
	KOH/%	-	0.1	0.15	0.1	0.1	0.15	0.1
	SDS/%	-	-	-	0.1	-	-	0.1
	LA/%	0.04	0.06	0.06	0.06	0.1	0.1	0.1
Tensile stress at 100% elongation/MPa		0.35	0.23	0.27	0.24	0.23	0.23	0.26
Tensile stress at 300% elongation/MPa		0.37	0.26	0.29	0.26	0.24	0.25	0.28
Tensile stress at 500% elongation/MPa		0.38	0.29	0.33	0.3	0.28	0.31	0.32
Tensile strength/MPa		1.45	0.68	1.05	0.77	0.66	0.75	0.9
Elongation at break/%		950	937	996	901	894	847	913
Tear strength/KN∙m^−1^		6.38	3.77	4.13	3.5	3.44	3.81	4.08
Hardness (shore)/°		13	8	9	7	7	6	7

**Table 5:** Stability and film formation of pre-vulcanized latex with different stabilization systems.

Stabilization Systems	MST /s	Heat Stability /s	Viscosity /MPa∙s^−1^	pH	Specific Conductance /μS∙cm^−1^	Film Appearance
0.7%NH_3_ + 0.04%LA	350	50	20	9.95	3781	Flat and shrinkage
0.1%KOH + 0.06%LA	425	51	52.5	8.15	4800	Smooth
0.15%KOH + 0.06%LA	500	23	98.4	8.31	5136	Relatively flat
0.1%KOH + 0.1%SDS + 0.06%LA	680	43	46.8	8.13	4767	Relatively flat
0.1%KOH + 0.1%LA	820	63	35.1	8.09	4829	A few wrinkles
0.15%KOH + 0.1%LA	880	32	55.6	8.30	5047	Relatively flat, with a few wrinkles
0.1%KOH + 0.1%SDS + 0.1%LA	>900	59	32.1	8.23	4823	Much wrinkles, uneven

**Table 6:** Vulcanization characteristics of CNRL compounded films with different stabilization systems.

Stabilization Systems	M_L_	M_H_	M_H_ − M_L_	T_10_/min	T_50_/min	T_90_/min
0.7%NH_3_ + 0.04%LA	2.0	3.18	1.18	1.01	7.22	25.87
0.1%KOH + 0.06%LA	1.64	3.19	1.55	0.25	4.82	26.16
0.15%KOH + 0.06%LA	1.59	3.19	1.6	0.33	5.54	26.40
0.1%KOH + 0.1%SDS + 0.06%LA	1.70	3.26	1.56	0.34	5.27	25.76
0.1%KOH + 0.1%LA	1.68	2.70	1.02	0.23	3.84	17.81
0.15%KOH + 0.1%LA	1.61	3.23	1.62	0.2	4.52	25.08
0.1%KOH + 0.1%SDS + 0.1%LA	1.66	2.98	1.32	0.25	3.97	21.74

**Table 7:** Physical and mechanical properties of CNRL vulcanized films with different stabilization systems.

Stabilization Systems	NH_3_/%	0.7	-	-	-	-	-	-
	KOH/%	-	0.1	0.15	0.1	0.1	0.15	0.1
	SDS/%	-	-	-	0.1	-	-	0.1
	LA/%	0.04	0.06	0.06	0.06	0.1	0.1	0.1
Tensile stress at 100% elongation/MPa		0.58	0.61	0.64	0.56	0.51	0.62	0.53
Tensile stress at 300% elongation/MPa		0.99	1.09	1.15	1.05	0.87	1.1	0.94
Tensile stress at 500% elongation/MPa		1.37	1.65	1.75	1.63	1.3	1.64	1.42
Tensile strength/MPa		25.94	21.68	25.45	26.64	16.36	23.07	18.3
Elongation at break/%		1156	1014	1019	1058	1080	1029	1013
Tear strength/KN∙m^−1^		51.04	54.78	53.44	42.89	40.27	44.31	46.98
Hardness (shore)/°		31.5	31	31	30.5	27.5	32	30.5

**Table 8:** Degradation temperature of CNRL dry films with different stabilization systems.

Stabilization Systems	T_0_/°C	T_50%_/°C	T_p_/°C	T_f_/°C
0.7%NH_3_ + 0.04%LA	352.7	381.1	383.6	414.6
0.15%KOH + 0.06%LA	353.1	376.5	384.6	413.4
0.1%KOH + 0.1%SDS + 0.06%LA	352.7	377.5	384.6	415.9
0.1%KOH + 0.1%LA	353.0	378.1	385.8	415.4

**Table 9:** Relative molecular weight of CNRL dry films with different stabilization systems.

Stabilization Systems	Mn/10^4^	Mw/10^4^	Distribution Coefficient
0.7%NH_3_ + 0.04%LA	29.52	124.14	4.21
0.15%KOH + 0.06%LA	28.23	122.25	4.33
0.1%KOH + 0.1%SDS + 0.06%LA	30.44	129.41	4.25
0.1%KOH + 0.1%LA	30.00	127.31	4.24

## Data Availability

Data will be made available on request.

## Abbreviations

NR	Natural rubber
NRL	Natural rubber latex
CNRL	Concentrated natural rubber latex
TD	Thioacetamide derivative
KOH	Potassium hydroxide
SDS	Sodium dodecyl sulfate
LA	Lauric acid
VFA number	Volatile fatty acid number
MST	Mechanical stability time
